# Sporotrichoid-Like Spread of Cutaneous* Mycobacterium chelonae* in an Immunocompromised Patient

**DOI:** 10.1155/2017/8219841

**Published:** 2017-08-23

**Authors:** Daria Marley Kemp, Anusha G. Govind, Jun Kang, Caroline C. Brugger, Young C. Kauh

**Affiliations:** ^1^Department of Dermatology and Cutaneous Biology, Thomas Jefferson University, Philadelphia, PA, USA; ^2^Department of Infectious Disease, Thomas Jefferson University, Philadelphia, PA, USA

## Abstract

*Mycobacterium chelonae* is a rapidly growing mycobacterium found in water and soil that can cause local cutaneous infections in immunocompetent hosts but more frequently affects immunocompromised patients. Typically, patients will present with painful subcutaneous nodules of the joints or soft tissues from traumatic inoculation. However, exhibiting a sporotrichoid-like pattern of these nodules is uncommon. Herein, we report a case of sporotrichoid-like distribution of cutaneous* Mycobacterium chelonae* in a patient with systemic lupus erythematosus on significant immunosuppressive medications. Clinicians treating immunocompromised patients should be cognizant of their propensity to develop unusual infections and atypical presentations.

## 1. Case Presentation

**Figure 1:**
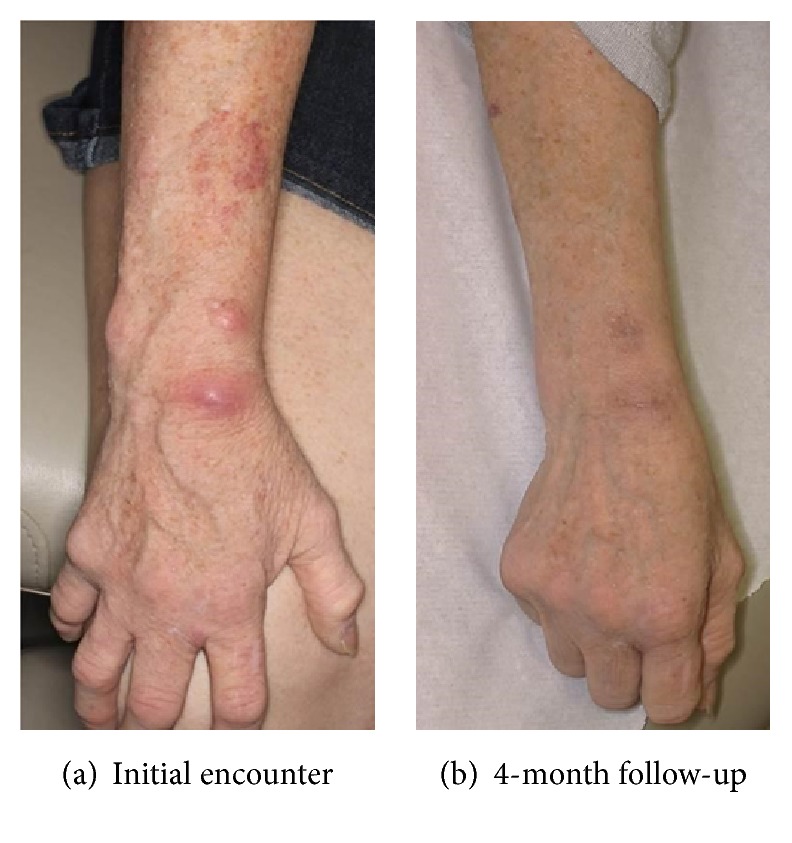
Clinical photos.

A 54-year-old female, with a history of systemic lupus erythematosus on mycophenolate mofetil (3 g daily), prednisone (10 mg daily), and cyclosporine (50 mg twice a day), presented with a 4-month duration of unilateral painful well-circumscribed erythematous to violaceous subcutaneous nodules extending from her 2nd finger web space to her dorsal wrist and forearm in a sporotrichoid-like pattern (Figure 1(a)). She denied exposure to fish tanks, swimming pools, tattoo needles, gardening, fresh or brackish waters, or nail salons.

**Figure 2:**
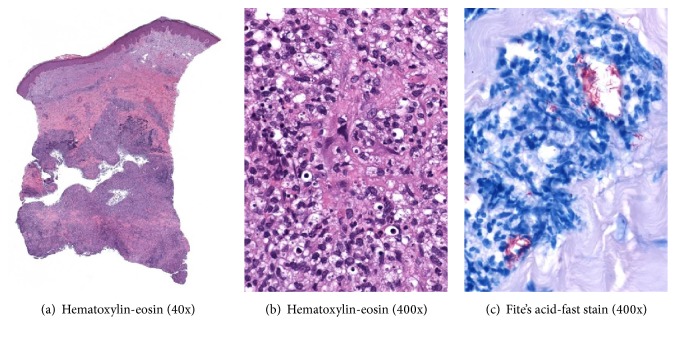
Pathologic findings from a right wrist skin nodule: suppurative granulomatous dermatitis in deep dermis (a & b) and numerous acid-fast bacilli highlighted by Fite's acid-fast stain (c).

Dorsal wrist nodule biopsy revealed suppurative granulomatous inflammation (Figures 2(a) and 2(b)). Both acid-fast bacilli (AFB) and Fite's acid-fast stains (Figure 2(c)) showed bacilli in the dermis, consistent with a mycobacterial infection. AFB culture and stain were positive for the rapid grower,* Mycobacterium chelonae*. Initially, she was started on dual antibiotic regimen of linezolid 600 mg twice daily and azithromycin 250 mg daily. Subsequently, she was converted to linezolid 600 mg and clarithromycin 250 mg twice daily once susceptibilities returned and also due to gastrointestinal upset from azithromycin. Although the patient had no adverse reaction to linezolid, the dose was decreased to 600 mg daily to ensure tolerability and continued normal blood counts. Given her active infection, rheumatology decreased her mycophenolate mofetil dose and discontinued cyclosporine. Improvement of her skin lesions was evident on follow-up within three months (Figure 1(b)). At four months, her nodules had fully resolved and therapy was discontinued.

## 2. Discussion

*Mycobacterium chelonae* is a rapidly growing mycobacterium commonly found in water and soil. The name* chelonae* is derived from the Greek word for turtle, chelōnē, as it was initially isolated in 1903 from sea turtles [1]. It can cause local cutaneous infections in immunocompetent patients, but infections are more frequently identified in patients taking immunosuppressive medications. Patients can develop painful subcutaneous nodules involving joints or soft tissue [2]. These skin nodules, as well as deeper and more disseminated infections, are usually from traumatic introduction. However, preceding trauma may not always be readily apparent. Sporotrichoid spread is a linear ascending extension along lymphatic chains, generally found with deep fungal infections but can also be present in other organisms. [3]. In 1992, Wallace et al. presented a series of 53 cases of* M. chelonae* disseminated cutaneous infections (>5 lesions), which were either well-circumscribed lesions or a “confluent mass of cellulitis with multiple draining fistulas.” They were generally at the distal end of an extremity but did not form a linear or sporotrichoid pattern [4]. Differential diagnosis for skin nodules in a sporotrichoid-spread pattern includes* Sporothrix schenckii, M. marinum*, other nontuberculous mycobacteria,* Nocardia*,* Leishmania*, and Tularemia [5].

Clinical presentation of* Mycobacterium chelonae* is often influenced by the immunological status of the patient. Approximately 10 cases have been described in the literature with a sporotrichoid-like pattern of* M. chelonae* involving an immunosuppressed patient [1, 3, 5–10] and at least two cases in a presumably immunocompetent patient [11]. Mycobacteria are referred to as AFB for the complex that forms during the histological staining process between the mycolic acid in the mycobacterial cell wall and dye, which is resistant to the decolorizing mineral acid. Compared to classic AFB staining, Fite's acid-fast stain uses a milder destaining acid, as well as less alcohol during the staining process, which it suitable for more delicate mycobacterium, such as* leprosy bacillus* [12].

Although clarithromycin was initially the drug of choice in treatment, increasing drug resistance makes it an unsuitable single agent. Treatment generally involves two antibacterial agents, particularly in immunocompromised patients. Linezolid has been shown to be an effective second medication; however, its adverse effects of bone marrow suppression warrant close monitoring [2]. Once daily dosing of linezolid 600 mg has demonstrated a potential benefit of decreasing the risk of toxicity over a prolonged course of treatment. However, a recent retrospective study found that the efficacy and safety profile of linezolid 600 mg once daily and 300 mg once daily in the treatment of multidrug and extensively drug-resistant tuberculosis showed potentially fewer neurotoxicities than a lower dose of linezolid [13].

Given the large number of immunocompromised patients clinicians serve, one must be mindful of their increased susceptibility to unusual infections and atypical presentations. It is vital that patients with suspicious sporotrichoid cutaneous lesions should have a biopsy with routine bacterial culture, mycobacterial culture, fungal culture, prolonged culture hold, molecular testing for* Nocardia*, and pathology. Only a few literature cases reported of* M. chelonae* present with sporotrichoid-like spread, typically in immunocompromised patients.

## Abbreviations and Acronyms

AFB: Acid-fast bacilli
*M. chelonae*: * Mycobacterium chelonae*
*M. marinum*: * Mycobacterium marinum*.
